# Olecranon stress fracture treated with headless compression screws and bone marrow aspirate concentrate augmentation: a case report and systematic review of the literature

**DOI:** 10.1016/j.xrrt.2025.06.014

**Published:** 2025-07-05

**Authors:** Austin Gartner, Collin Freking, Vafa Behzadpour, William G. Messamore

**Affiliations:** Department of Orthopedic Surgery, The University of Kansas School of Medicine, Wichita, KS, USA

**Keywords:** Olecranon stress fracture, Headless compression screw, Headless screw, Olecranon, Elbow injury, Surgical management

## Abstract

**Background:**

Several surgical techniques have been described to treat olecranon stress fractures (OSFs), most commonly involving the use of cannulated screws placed perpendicular to the fracture line of the olecranon. However, hardware removal, infection, and pain are common. This study presents a novel surgical approach and investigates the current literature surrounding the use of headless compression screws (HCSs) in the surgical management of OSFs.

**Methods:**

A search of PubMed, Embase, Cochrane, CINAHL, MedLine, and SportDiscus was performed by three reviewers using search terms “Screw” AND “Olecranon stress fracture,” following the Preferred Reporting Items for Systematic Reviews and Meta-Analyses guidelines. Inclusion criteria consisted of studies that focused on surgical treatment of OSFs and discussed the use of HCSs. Articles were excluded if they did not discuss OSFs, discussed surgical treatment with non–HCSs, did not have full text available, or were written in a non-English language.

**Results:**

Sixty-two studies met the search criteria, of which 3 studies met inclusion and exclusion criteria. Across the included studies, 12 throwing athletes were treated for OSFs with HCSs, with a return-to-sport time between 4-6 months (mean, 5.5 months). No occurrences of hardware infection were identified, nor was removal of hardware required in any instances.

**Conclusion:**

Current literature demonstrates promising results for the use of HCSs in the setting of OSFs. Future prospective studies are needed to compare surgical outcomes and rates of symptomatic hardware to traditional fixation methods.

An olecranon stress fracture (OSF) is an uncommon phenomenon in the context of sports injuries, with a prevalence of 5.4% among baseball-related elbow disorders.^6^ This injury was first described by Waris et al in 1946, detailing a case in javelin throwers,^18^ but it was not until 1968 that the OSF was described in baseball players.^16^ OSFs in baseball players are primarily a result of valgus extension overload forces, including the olecranon abutting the fossa, triceps traction on the olecranon during the deceleration phase of throwing, and medial impact secondary to valgus stress.^17^

Management of OSFs is guided by classification of the stress fracture based on the five-stage radiographic differentiation system (Supplementary Appendix S1) described by Furushima et al, with further differentiation into four stages for physeal OSFs (Supplementary Appendix S2). It was suggested that “surgery is indicated in physeal (stages 3 and 4), classic, transitional, and distal types after approximately three months of nonoperative treatment.”^6^ However, strong literature supporting when to pursue conservative vs. surgical treatment is still lacking.

A variety of surgical techniques have been described to treat OSFs, most commonly involving the use of cannulated screws placed perpendicular to the fracture line of the olecranon.^4^ However, regardless of the surgical methodology used, hardware removal due to mechanical failure, infection, and pain is commonplace. Smith et al reported an overall complication rate of 22.5% with operative management of OSFs, with symptomatic or infected hardware comprising the majority of complications.^17^ Paci et al postulated that the subcutaneous nature of the screw head after fixation may increase infection risk.^12^ To address this, we present a novel surgical approach using open reduction and internal fixation (ORIF) with two headless compression screws (HCSs), augmented with bone marrow aspirate concentrate (BMAC)–treated cancellous allograft bone, to avoid complications associated with large, prominent compression screws. The patient was informed, and consent was obtained to publish details about the case, including imaging. A systematic review was then completed to evaluate the use of HCSs in the surgical management of OSFs to better understand the current literature available on this topic.

## Systematic review

A systematic review was performed regarding current surgical management of OSFs. More specifically, the objective was to determine whether previous literature exists regarding the use of HCSs for the treatment of OSFs.

## Methods

A comprehensive literature search was performed following the guidelines directed by Preferred Reporting Items for Systematic Reviews and Meta-Analyses. Utilizing 6 separate online databases (PubMed, Embase, Cochrane, CINAHL, MedLine, and SportDiscus), a search was performed by three reviewers using search terms “Screw” AND “Olecranon stress fracture.” The databases were searched from inception to the original search date, February 22, 2024. After search results were obtained, duplicates were removed. Articles were screened by reviewing the full-text manuscript. When disagreements occurred, the articles were reviewed by the three reviewers, and a final decision was made for each article. The full text was then reviewed, and our predetermined inclusion and exclusion criteria were applied. Inclusion criteria consisted of (1) studies that focused on surgical treatment of OSFs, and (2) studies that discussed the use of HCSs. Articles were excluded if they (1) did not discuss OSFs, (2) discussed surgical treatment with non–HCSs, (3) did not have full text available, and were (4) written in a non-English language.

## Results

**Figure 1:**
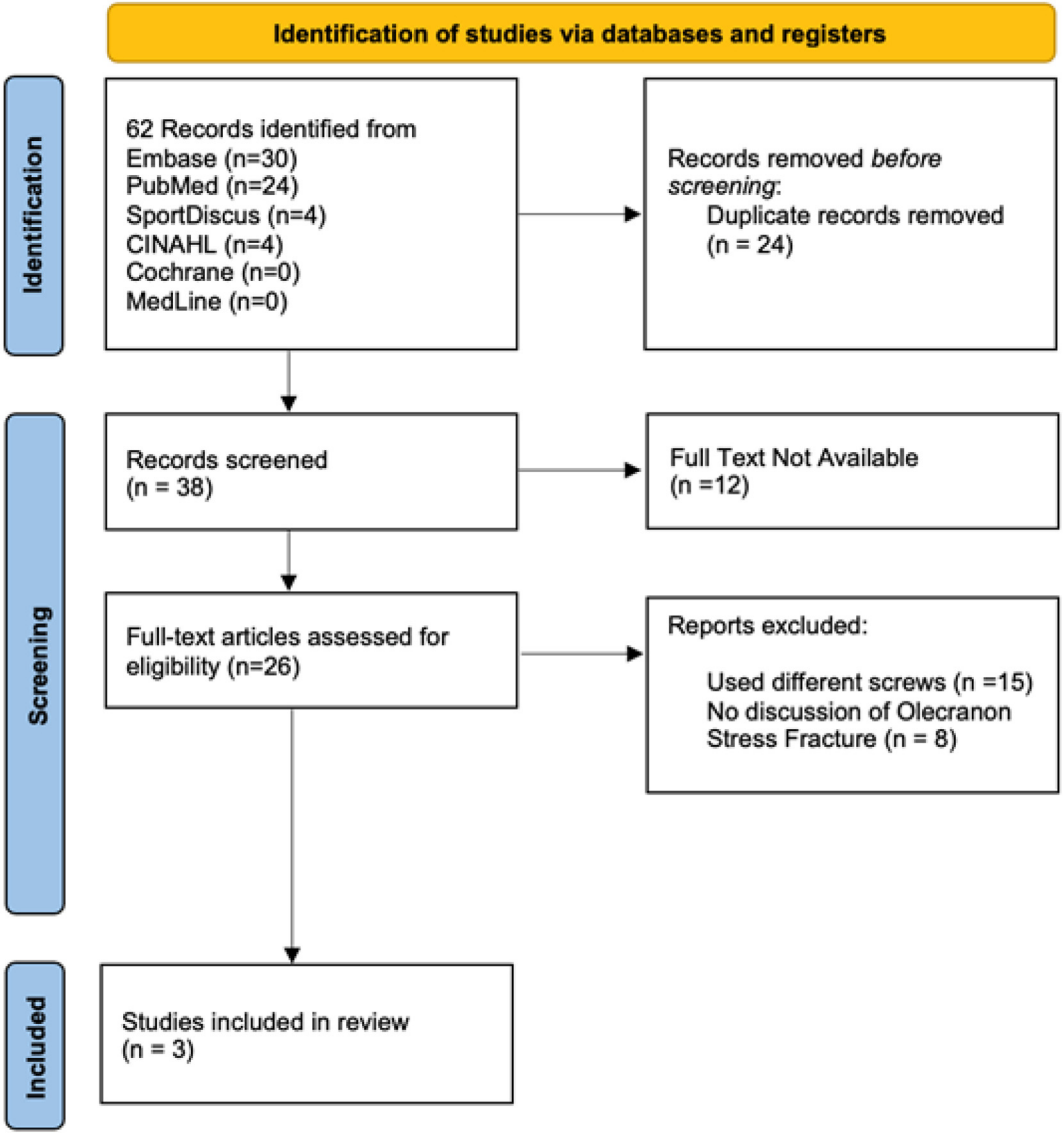
PRISMA flowchart for studies included in the systematic review. *PRISMA*, Preferred Reporting Items for Systematic Reviews and Meta-Analyses.

Sixty-two studies were identified that met search criteria. Twenty-four duplicate articles were identified and removed from the analysis. The remaining 38 studies were screened, of which 12 were removed that did not contain full-text articles. Twenty-six studies were assessed for eligibility, and 3 studies met inclusion and exclusion criteria (Fig. 1). Characteristics of the studies included are listed in Table I.

**Table I:** Characteristics of included studies.

First author	Number of patients	Average age	Treatment	Sport	Time to return-to- sport
Fujioka^5^	6	18	ORIF with Double Threaded screw Japan, Meira, Japan large screw (cannulated double-threaded headless screw)	5 baseball, 1 softball	6 mo
Michelin^11^	5	20	ORIF with two headless compression screws	5 baseball	5.8 mo
Schwab^15^	1		Arthroscopic removal of posteromedial osteophytes and arthroscopic-assisted screw fixation with a cannulated headless compression screw		4-5 mo

*ORIF*, open reduction and internal fixation.

Across the 3 included studies, 12 throwing athletes were treated for OSFs with HCSs either after unsuccessful conservative treatment or due to a desire for expedited return to high-level play. All athletes returned to their throwing sport between 4 and 6 months postsurgery, with the average time to return being 5.5 months. Fujioka et al examined six throwing athletes who underwent ORIF with headless cannulated double-threaded screws following weeks to months of reduced sports activity but persisting elbow pain.^5^ Full range of motion (ROM) was obtained, and a gradual throwing program was initiated at 4-6 weeks postsurgery for each patient. Follow-up demonstrated full return to competitive-level throwing by 6 months, with no pain or ROM loss.

Another study, Michelin et al, examined five competitive throwing athletes who underwent ORIF with two HCSs with the goal of expediting return to play for these high-level throwing athletes.^11^ Patients achieved pain-free return to sport at a mean of 5.8 months postsurgery, and no complications were reported at the 17-month follow-ups. Lastly, Schwab et al discussed a case of an individual who underwent arthroscopy for posteromedial impingement and fixation of an OSF with a single cannulated fully threaded HCS.^15^ The patient returned to a throwing program after 12 weeks and competitive play 4-5 months after surgery. No occurrences of hardware infection were identified in any of the included studies, nor was removal required in any instances.

## Case report

**Figure 2:**
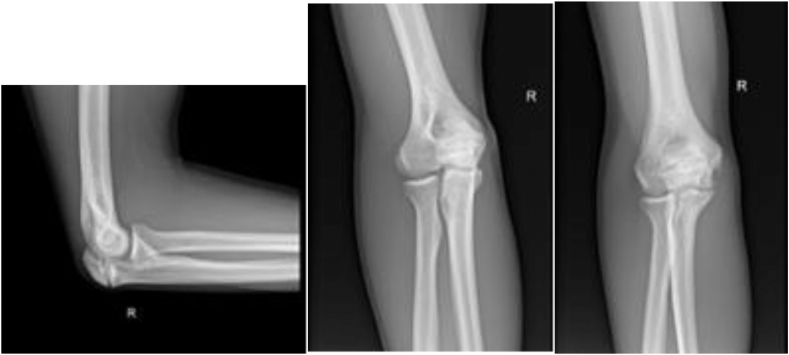
Initial injury plain films: transverse olecranon stress fracture with sclerotic fracture edges. Well-maintained ulnohumeral and radiocapitellar joint spaces.

**Figure 3:**
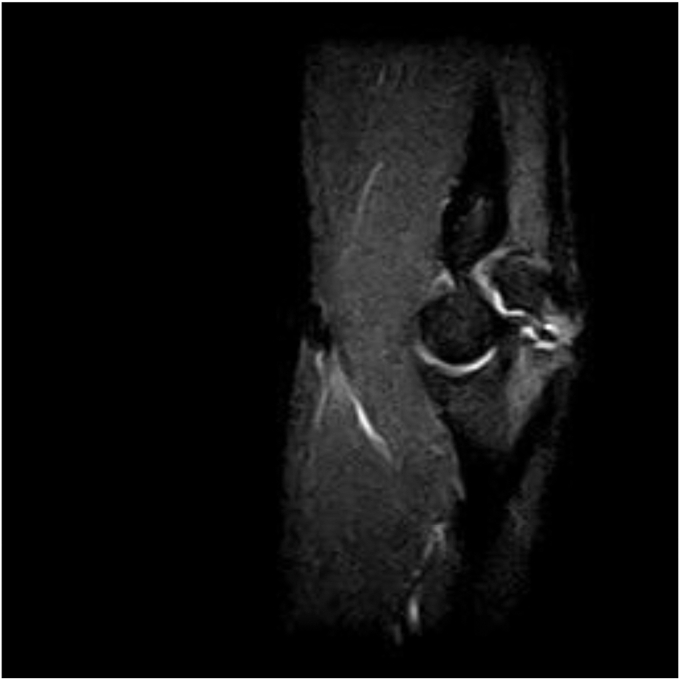
Initial injury MRI: MRI demonstrates olecranon stress fracture with cortication at the fracture site with rounded margins. No bony bridging present at the fracture site and accompanied marrow edema is present. *MRI*, magnetic resonance imaging.

A twenty-one-year-old right-handed Caucasian collegiate baseball player with no medical history presented from an outside clinic with persistent right medial elbow pain along with numbness and tingling in the ring and small fingers while throwing. An ulnar collateral ligament injury was initially suspected; multiple months of conservative treatment with throwing cessation, scapular stabilization, and rehabilitation did not ameliorate the pain. On physical examination, the patient had full passive and active elbow flexion and extension. He was tender to palpation over the flexor–pronator mass as well as the medial aspect of the elbow along the ulnar collateral ligament and had pain with resisted pronation and elbow flexion. Radiographs (Fig. 2) and magnetic resonance imaging (Fig. 3) were obtained, revealing a type 2 OSF. Magnetic resonance imaging demonstrated OSF with cortication at the fracture site with rounded margins. No bony bridging was present at the fracture site, and accompanied marrow edema present.

Due to the chronicity of right elbow pain from OSF and unsuccessful conservative management, operative intervention was suggested to expedite the athlete's return to competition. Twenty-four days after the initial visit, the patient underwent an ORIF using two 3.5-mm HCSs (Arthrex, Naples, FL, USA) and cancellous bone allograft treated with iliac BMAC at the fracture site.

### Description of procedure

Under general anesthesia, a 2-mm incision was placed 4 cm proximal to the anterior superior iliac spine along the iliac crest. A Jamshidi needle was inserted through the iliac crest, and 60 cc of bone marrow was aspirated and centrifugated into BMAC.^3^ Five cc of demineralized bone allograft was then soaked in the BMAC while the fracture site was prepared. Aspiration of bone marrow and soaking of bone allograft was preferred due to perceived decrease in morbidity with similar union rates in comparison to iliac crest autograft.^10^ An 8-cm incision was made over the patient's olecranon with efforts to prevent incision right over the tip of the olecranon. Dissection of skin and subcutaneous tissue was carried down to the olecranon where the fracture was identified. After removal of fibrous tissue and exposure of the fracture site, sclerotic bone from the proximal and distal fragment was removed with a burr. The BMAC-treated allograft bone was then placed in the fracture site prior to fracture reduction and compression. Two 2-mm drill holes were placed distal to the fracture site, allowing for reduction clamps to secure and compress the fracture. The reduction was confirmed via fluoroscopy. Guide wires were placed across the fracture, and two 3.5-mm HCSs, lengths 40 mm and 44 mm, were then placed across the fracture site, providing excellent compression of the fracture. Intraoperative examination confirmed full ROM, and the incision was subsequently closed in a layered fashion. The patient was placed in a posterior slab splint and sling and instructed to be nonweight-bearing on the right upper extremity.

### Follow-up

One week postoperatively, the patient had full passive ROM with intact sensation and motor function and moderate swelling of the elbow. Radiographs demonstrated satisfactory compression across the fracture site with no acute osseous abnormalities. The patient was transitioned out of his splint and started physical therapy with active-assisted ROM. The patient was started on vitamin D, potassium, magnesium, and calcium supplementation to aid in fracture consolidation. Magnesium was added to the standard bone-support supplements—vitamin D, potassium, and calcium—because of postulated benefits in fracture healing, resulting from proven benefits of bone mineral density and decreased fracture risk.^14^

**Figure 4:**
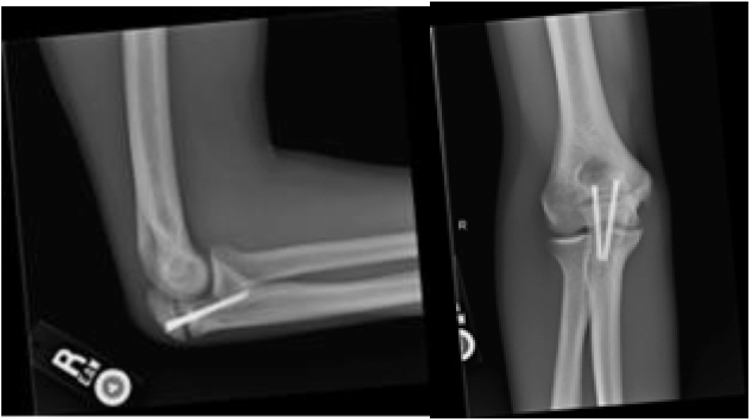
4-week postoperative plain films: persistent transverse olecranon fracture nonunion with two well-aligned headless compression screws and resorption of bone graft.

One month postoperatively, physical examination demonstrated healed surgical incisions. Active ROM demonstrated full flexion, missing terminal extension by 3-5 degrees. The patient stated that he only appreciated pain on terminal extension of the elbow. Radiographs demonstrated some resorption of the bone graft with minimal bridging bone (Fig. 4).

**Figure 5:**
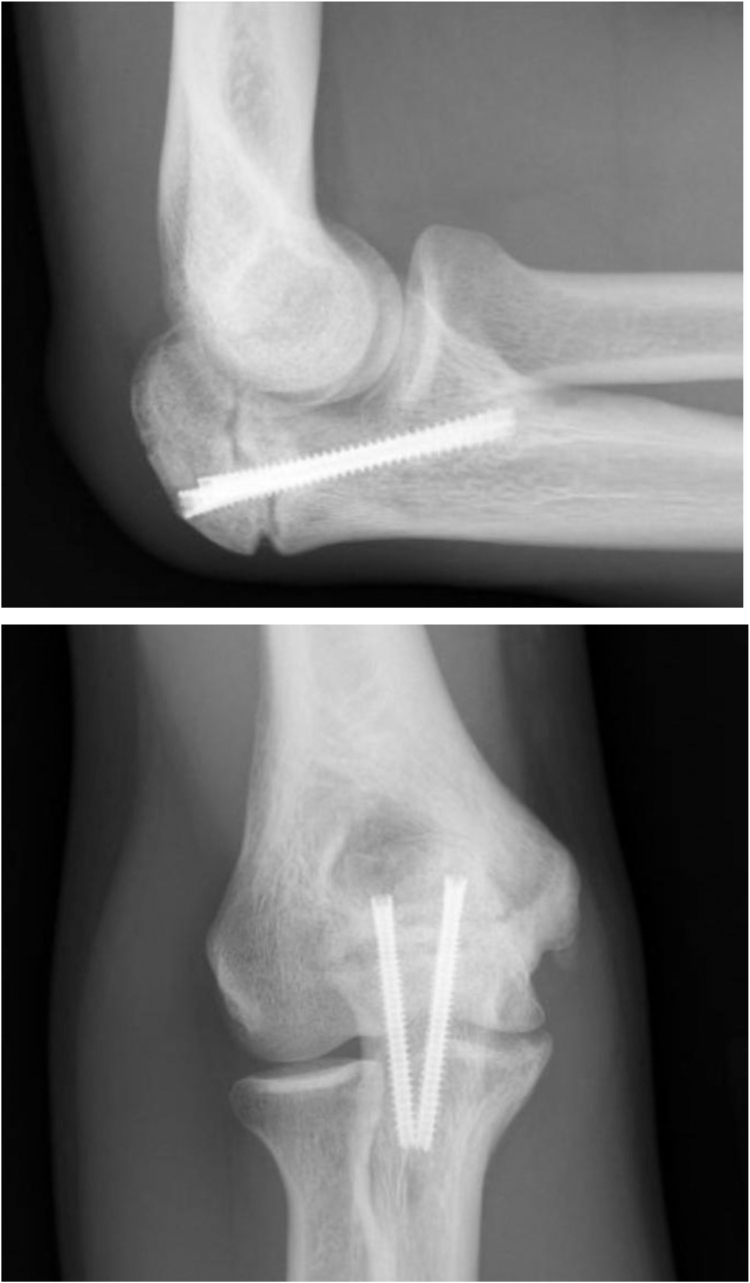
3-month postoperative plain films: well-maintained alignment of hardware with minimal callus formation and persistent nonunion of fracture edges.

Three months out from surgery, the patient initiated a light resistance program with minimal pain. Physical exam displayed full extension and flexion. Palpation along both the olecranon and medial aspect of the elbow did not reproduce any pain. The patient maintained full pronation and supination, 5/5 strength with elbow flexion, extension, supination, and pronation. Bounce test and moving dynamic valgus test were both negative. Radiographs depicted an increase in bridging callus compared to prior films (Fig. 5). After confirmation of the bridging callus, the patient was placed in a collegiate return-to-throwing program and instructed to progress activity as tolerated.

**Figure 6:**
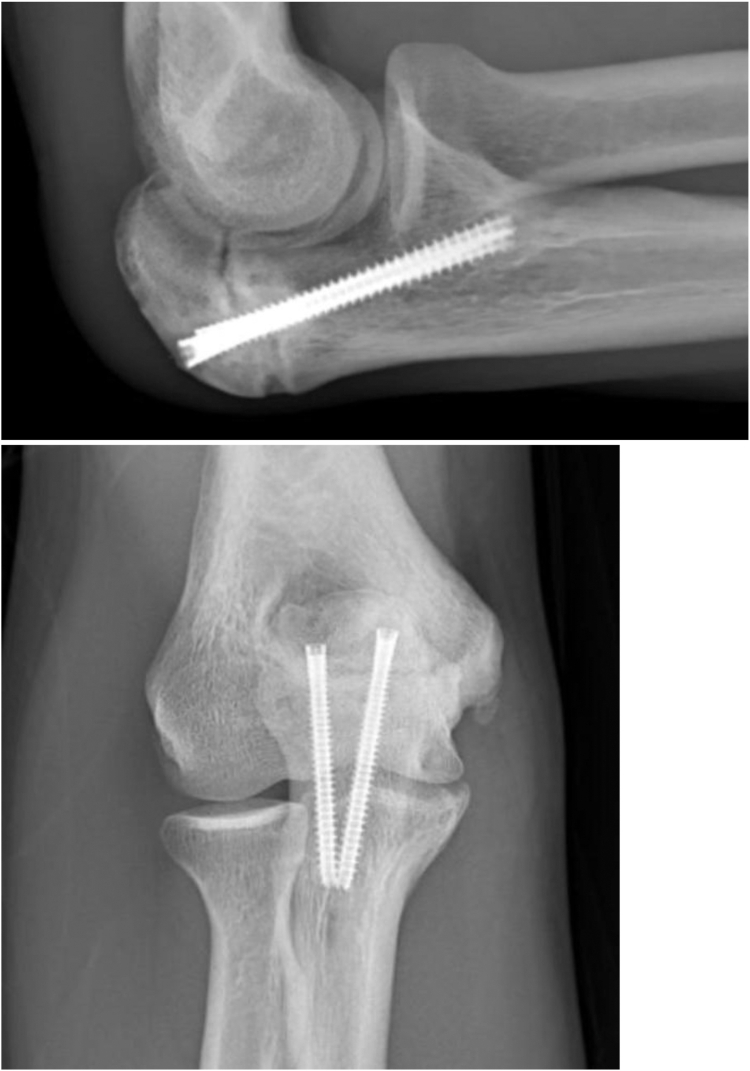
7-month postoperative plain films: continued well-maintained alignment of hardware with improved moderate callus formation and persistent sclerotic fracture edges.

At 7 months postoperatively, physical examination exhibited painless, full ROM with 5/5 strength in all elbow movements. Dynamic valgus and bounce tests were negative. Radiographs demonstrated complete bony bridging and bony consolidation across the previous OSF site (Fig. 6). Patient was released from clinic at this time without any restrictions with return to prior level of play. Final Single Assessment Numeric Evaluation score of 100.

At 2 years postoperatively, the patient remains asymptomatic. He maintains full ROM and returned to collegiate-level throwing without pain.

## Discussion

First-line treatment for OSFs is typically conservative management, consisting of rest, immobilization, and cessation of throwing. In the case of failure of nonoperative management, true fracture, or apophyseal nonunion, OSFs can be treated with ORIF with compression screws or plate osteosynthesis.^17^ While ORIF remains the gold standard surgical approach, current methods are marked by significant complication rates. Paci et al revealed that out of 18 patients treated with ORIF using single cannulated titanium screws, 10 (56%) required additional surgeries on their throwing arm, with 6 having hardware-related complications.^12^ Another systematic review by Smith et al found a 22.5% complication rate among 40 patients undergoing operative management, with 20% involving infected hardware.^17^

The high rates of complication and hardware removal, especially in the setting of nondisplaced fractures, have led to an investigation of novel surgical approaches. The utilization of HCSs offers several advantages. Michelin et al notes that HCSs allow for a reduction in violation of the triceps tendon.^11^ In addition, this surgical methodology may decrease risk of hardware irritation and future removal, which is cited to be as high as 33%-40% in this population. Michelin's study consisted of five patients who underwent treatment with retrograde cannulated HCSs. All patients were able to return to the same level of play or higher at a mean of 5.8 months, with no incidence of hardware removal. The results presented in the current study confirm those of Michelin's study and demonstrate that this technique is both safe and effective for getting athletes back to play quickly without any of the aforementioned hardware complications that have been noted in the literature.

There was initial concern with comparison of the level of interfragmentary compression that can be achieved with headless screws compared to their headed counterparts. Traditional screw types have the ability to provide compression on the proximal fragment as it exerts a “buttress” effect with its head; however, HCSs do not have this luxury.^9^ Previous biomechanical studies evaluated these characteristics in fractures of the hand, namely scaphoid fractures. Gruszka et al analyzed three new-generation compression screws, including the Acutrak 2 Mini (Acumed, Hillsboro, OR, USA), Stryker TwinFix (Stryker, Kalamazoo, MI, USA), and Synthes 3.0 mm HCSs (DePuy Synthes, Raynham, MA, USA). Compressive forces immediately after screw insertion were all substantially greater than the Synthes 2.0 mm cortical lag screw (DePuy Synthes, Raynham, MA, USA) that was used as a reference.^7^ Pensy et al found similar results with the Synthes 3.0-mm and Acutrak standard compression screw.^13^

In another study, Kozakiewicz et al evaluated compressive forces of four different modified HCSs with that of titanium lag screws for use in mandible head fracture treatment. It was found that the headless screws lost only a small, clinically insignificant amount of compressive ability in comparison to that of the lag screw. Kozakiewicz additionally noted that the advantageous nature of the nonirritant screw construction, along with the headless screw's mechanical properties, make it an attractive option for condylar head fracture treatment.^9^

These compressive forces have been shown to be maintained across various insertion depths. Hart et al analyzed the compressive forces and insertion torque of four different headless screws at a variety of insertion depths. Each of the screws demonstrated at least 60 N of compressive force throughout the range of insertion depths. Notably, the depth at which peak compression occurred varied significantly among the screws (−3.1 mm, −2.8 mm, 0.9 mm, and 1.5 mm).^8^ These findings suggest that, despite the initial concerns regarding the level of interfragmentary compression of headless screw designs, it may not be a limiting factor in the decision to use these screws.

The limited studies available show promising results with use of HCSs in the setting of OSFs. Fujioka et al reported six patients who underwent ORIF with headless, double-threaded, cannulated screws with no removal, pain, loss of ROM, and were able to return to competitive baseball.^5^ The case series by Fujioka et al was limited to stress fractures not seen on normal radiographic films and required a CT scan for diagnosis. This lack of significant sclerotic bone at the fracture site allowed for treatment without exposure of the fracture site.^5^ Michelin et al reported five competitive throwing athletes who underwent ORIF with two HCSs.^11^ No complications were reported within 17 months of follow-up, and patients returned to sport at a mean of 5.8 months postsurgery. Schwab et al describes a case of a baseball player who underwent fixation of an OSF with a single, cannulated, fully threaded HCS who was able to return to throwing after 12 weeks and competitive play at just over 4 months without complication.^15^

In the present case report, the twenty-one-year-old collegiate baseball player had undergone conservative therapy for months without improvement and resorted to surgical management with hope for an expeditious return to competition. This case was unique in its use of two 3.5-mm Arthrex HCSs and cancellous bone allograft treated with iliac BMAC along the fracture site. This patient regained full, pain-free ROM and was able to return to a throwing program three months postoperatively, with return to competition at about seven months postoperatively. This return-to-sport timeline is similar to those described in the previous included studies.

No studies specific to OSFs have compared hardware removal rates between screw types. However, other studies have shown that headless screws reduce soft-tissue irritation in calcaneal osteotomies^1^; and in the treatment of femoral neck fractures, headless cannulated screws had a removal rate of 11%, compared with 47% for headed screws.^19^ These findings suggest a potential advantage of headless screw designs in minimizing the need for subsequent hardware removal. While HCSs are known to be relatively more expensive compared to standard screws, their minimized need for subsequent hardware removal may present an overall cost benefit on the health care system.^2^ Further studies are needed comparing the rates of symptomatic hardware of HCSs with other OSF fixation methods.

This present study is not without limitations. Limited sample size of the studies included in the systematic review impacts the generalizability of the results. Much of the data on the utility of HCSs has been seen in other surgical applications. In addition, the included studies were neither randomized controlled trials nor prospective study design. Lastly, 2-year postop films were unable to be obtained, as the patient moved out of state and has since been lost to follow-up.

## Conclusion

The use of BMAC with cancellous allograft in the setting of two HCSs is a novel technique for treating OSFs. Current literature shows promising early results for use of HCS in the setting of OSFs; however, future prospective studies are needed comparing outcomes to traditional fixation methods as well as rates of symptomatic hardware and subsequent need for hardware removal.

## Disclaimers:

Funding: No funding was disclosed by the authors.

Conflicts of interest: The authors, their immediate families, and any research foundation with which they are affiliated have not received any financial payments or other benefits from any commercial entity related to the subject of this article.
