# Supplementary material for: Olecranon stress fracture treated with headless compression screws and bone marrow aspirate concentrate augmentation: a case report and systematic review of the literature
Source: JSES Rev Rep Tech. 2025 Jul 5;5(4):976–83. doi: 10.1016/j.xrrt.2025.06.014 (PMC12573636; doi:10.1016/j.xrrt.2025.06.014)
Supplement: Supplementary Appendix S1 [file mmc1.docx]

Appendix A: Furushima et al.’s Classification of OSFs

| Fracture Type | Description |
| --- | --- |
| Physeal | Delayed closure or nonunion is found along the line of the epiphyseal plate |
| Classic | Fracture line originates from the proximal-ulnar side and runs toward distal-radial side |
| Transitional | In line with the epiphyseal plate, running vertical to the ulnar axis on AP radiographs |
| Sclerotic | Fracture line is not obvious on plain radiographs, but sclerotic bone is seen |
| Distal | Fracture line originates from cortical notch of the trochlear groove |
