# Supplementary material for: Olecranon stress fracture treated with headless compression screws and bone marrow aspirate concentrate augmentation: a case report and systematic review of the literature
Source: JSES Rev Rep Tech. 2025 Jul 5;5(4):976–83. doi: 10.1016/j.xrrt.2025.06.014 (PMC12573636; doi:10.1016/j.xrrt.2025.06.014)
Supplement: Supplementary Appendix S2 [file mmc2.docx]

Appendix B: Classification of Physeal Olecranon Stress Fractures

| Stages of Olecranon Stress Fractures | Characteristics of the Epiphyseal Plate. |
| --- | --- |
| Stage 1 | Epiphyseal plate on nonthrowing side is closed incompletely; epiphyseal plate on throwing side is delayed |
| Stage 2 | Epiphyseal plate on nonthrowing side is closed completely, but is not closed on throwing side |
| Stage 3 | Epiphyseal plate dehiscence observed on articular surface |
| Stage 4 | Complete dehiscence of the epiphyseal plate is seen from the articular surface to the dorsal olecranon |
